# Study protocol on the role of intestinal microbiota in colorectal cancer treatment: a pathway to personalized medicine 2.0

**DOI:** 10.1007/s00384-017-2819-3

**Published:** 2017-04-25

**Authors:** R. Aarnoutse, J. M. P. G. M. de Vos-Geelen, J. Penders, E. G. Boerma, F. A. R. M. Warmerdam, B. Goorts, S. W. M. Olde Damink, Z. Soons, S. S. M. Rensen, M. L. Smidt

**Affiliations:** 1 GROW-School for Oncology and Developmental Biology, Maastricht University Medical Centre, Universiteitssingel 40, 6229 ER Maastricht, the Netherlands; 2 Department of Surgery, Maastricht University Medical Centre, P. Debyelaan 25, 6229 HX Maastricht, the Netherlands; 3 Department of Medical Oncology, Maastricht University Medical Centre, P. Debyelaan 25, 6229 HX Maastricht, the Netherlands; 4 Department of Medical Microbiology, Maastricht University Medical Centre, P. Debyelaan 25, 6229 HX Maastricht, the Netherlands; 5 Department of Surgery, Zuyderland Medical Centre, Dr. H. van der Hoffplein 1, 6162 BG Geleen, the Netherlands; 6 Department of Medical Oncology, Zuyderland Medical Centre, Dr. H. van der Hoffplein 1, 6162 BG Geleen, the Netherlands; 7 NUTRIM - School of Nutrition and Translational research In Metabolism, Maastricht University Medical Centre, Universiteitssingel 40, 6229 ER Maastricht, the Netherlands

**Keywords:** Intestinal microbiota, Colorectal cancer treatment

## Abstract

**Purpose:**

Investigate in patients with metastatic and/or irresectable colorectal cancer treated with systemic treatment with capecitabine or TAS-102 whether:

1. Intestinal microbiota composition can act as a predictor for response.
2. Intestinal microbiota composition changes during systemic treatment and its relation to chemotoxicity.

**Backgro**u**nd:**

Gut microbiota and host determinants evolve in symbiotic and dependent relationships resulting in a personal ecosystem. In vitro studies showed prolonged and increased response to 5-fluorouracil, a fluoropyrimidine, in the presence of a favorable microbiota composition. Capecitabine and TAS-102 are both fluoropyrimidines used for systemic treatment in colorectal cancer patients.

**Methods:**

An explorative prospective multicenter cohort study in the Maastricht University Medical Centre+ and Zuyderland Medical Centre will be performed in 66 patients. Before, during, and after three cycles of systemic treatment with capecitabine or TAS-102, fecal samples and questionnaires (concerning compliance and chemotoxicity) will be collected. The response will be measured by CT/MRI using RECIST-criteria. Fecal microbiota composition will be analyzed with 16S rRNA next-generation sequencing. The absolute bacterial abundance will be assessed with quantitative polymerase chain reaction. Multivariate analysis will be used for statistical analysis.

**Conclusions:**

We aim to detect a microbiota composition that predicts if patients with metastatic and/or irresectable colorectal cancer will respond to systemic treatment and/or experience zero to limited chemotoxicity. If we are able to identify a favorable microbiota composition, fecal microbiota transplantation might be the low-burden alternative to chemotherapy switch in the future.

## Background

The interest in intestinal microbiota in relation to cancer is rapidly growing. The human gastro-intestinal tract contains over 1 kg bacteria. The collective genome of all these bacteria is called the microbiome. The intestinal microbiota exerts crucial functions that humans cannot exert themselves while human hosts offer a nutrient-rich environment. In healthy people, a dynamic equilibrium exists. However, environmental factors and host genetic factors all can influence microbiota composition and generate dysbiosis related to carcinogenesis and disturbed metabolism [1–3]. Microbiota differences were reported between healthy people and patients with colorectal and gastric cancer [4, 5]. Especially in gastric cancer, the presence of *Helicobacter pylori* is highly associated with cancer development [4]. A recent study showed that the human intestinal microbiota signature is able to predict in vivo treatment response to antibiotics in patients with *Clostridium difficile* infections [6]. Another in vivo study used microbiota composition of the female urogenital tract to predict failure of implementation of an embryo in in vitro fertilization (IVF) [7]. The pilot study results of the Erasmus Medical Center showed that IVF fails with 96% certainty in the presence of an unfavorable microbiota profile [8].

Colorectal cancer (CRC) is the third most common cancer in the world. The standard systemic treatment in CRC in metastatic and/or irresectable setting is mostly based on fluoropyrimidine, such as 5-fluorouracil (5-FU), orally administered capecitabine, or TAS-102. Capecitabine is a precursor of 5-FU [9]. Capecitabine is frequently administered in combination with bevacizumab, which is an antibody that binds to the vascular endothelial growth factor (VEGF). Bevacizumab inhibits angiogenesis and its related tumor growth. TAS-102 consists of cytotoxin trifluridine and thymidine phosphorylase inhibitor (TPI) tipiracil. Tipiracil prevents rapid metabolism of trifluridine, increasing the bioavailability of trifluridine, which inhibits tumor cell growth [10].

Response to systemic treatment is often defined as complete or partial response, stable, or progressive disease. Only 10–15% of the advanced CRC patients actually respond positively to the administration of 5-FU alone. Furthermore, patients treated with the fluoropyrimidine capecitabine frequently experience chemotoxicity with grades 3–4 chemotoxicity diarrhea of 5%, 2% nausea or vomiting, 4% mucositis, 3% anemia, and 1% hand-foot syndrome [9, 10]. Response to a systemic therapy also depends on patient’s dihydropyrimidine deficiency (DPD) status [11]. DPD is a molecular determinant of capecitabine efficacy in CRC patients [12]. It is known that patients with DPD experience more chemotoxic effects, since dihydropyrimidine cannot process thymine and uracil [13]. Therefore, patients DPD status will be standardly determined in an increasing number of hospitals.

There is little evidence in the field of CRC (treatment) and the role of human intestinal microbiota composition. Existing evidence is limited to in vitro studies. One in vitro study recently indicated a better response of CRC cell lines to 5-FU in the presence of *Lactobacillus plantarum* supernatant [14]. Another in vitro study indicated that 5-fluorocytosine (5-FC) (structurally related to 5-FU) samples, incubated with viable *Escherichia coli* showed a higher increase in active 5-FU concentration compared to samples incubated with non-viable *E*. *coli*. When 5-FC was incubated with human fecal samples, a significant degradation reaction of 5-FC was observed when compared to samples that were incubated with human feces that received an antimicrobial treatment. These in vitro studies indicate better responses in the presence of human intestinal bacteria [15, 16].

Thus, in the field of CRC treatment, no studies currently bridge the high potential translational gap between previous promising (in vitro) studies results and clinic. This study aims to bridge this translational gap in order to explore the importance of the human intestinal microbiota in CRC treatment by initiating an explorative prospective multicenter cohort study in the Maastricht University Medical Centre and Zuyderland Medical Centre.

If the human microbiota can predict response to systemic treatment with a non-invasive method, chemotoxicity can be reduced in non-responders and/or systemic therapy regimes can be changed. Further, change of the intestinal microbiota related to response and chemotoxicity can also help to identify with a non-invasive method “the favorable microbiota composition”. In case of inflammatory bowel syndrome and *C*. *difficile* infection, fecal microbiota transplantations are already performed. If fecal microbiota transplantations could be applied in order to increase and prolong response to systemic treatment with limited chemotoxicity, wouldn’t that be an impressive step forward?

In conclusion, the field of research of the human microbiota is new. By further exploring the intestinal microbiota composition before and after systemic treatment in patients with metastatic and/or irresectable CRC, an area of personalized medicine 2.0 can be created resulting in an increased response rate and improved chemotoxicity profiles. In the future, new therapeutic pathways could be created by providing fecal microbiota transplantation to replenish, maintain, or create an optimal microbiota composition [17].

## Methods

### Study objectives

#### Primary objective

The primary object is to investigate whether the microbiota composition can act as a predictor for response and/or chemotoxicity to three cycles of systemic treatment with capecitabine (with or without bevacizumab) or TAS-102 in patients with metastatic and/or irresectable CRC.

#### Secondary objective

The secondary objective of this study is to investigate the microbiota composition changes during systemic treatment with capecitabine (with or without bevacizumab) or TAS-102 and its relation to response and/or chemotoxicity in patients with metastatic and/or irresectable CRC.

### **Study design**

An explorative multicenter cohort study will be performed in the Maastricht University Medical Centre+ (MUMC+) and Zuyderland Medical Centre.

### Population

All patients with metastatic and/or irresectable CRC who will receive three cycles systemic treatment are eligible to participate in this study. Systemic treatment can consist of either oral capecitabine (with or without intravenous bevacizumab) or oral TAS-102. This patient group is selected, since these patients receive only one chemotherapeutic agent, and this will optimize homogeneity. To further optimize the homogeneity, patients with microsatellite instability (MSI) will be excluded. The patients visiting the outpatient clinic of the MUMC+ or Zuyderland Medical Centre can be selected for participation.

### Inclusion criteria

- Patients diagnosed with metastatic and/or irresectable CRC who will be treated with oral capecitabine (with or without intravenous bevacizumab) or oral TAS-102
- Aged 18 years or older
- Written informed consent

### Exclusion criteria

- Microsatellite instability (MSI)
- Has not received any prior systemic therapy for the treatment of CRC during the previous 4 weeks prior to start the current line of capecitabine or TAS-102
- Patients treated with additional systemic treatments during planned treatment period
- Radiotherapy within 2 weeks
- Therapeutic antibiotic use within past 3 months
- Renal function: calculated creatinine clearance (Cockroft-Guilt) < 30 ml/min
- Pregnant or nursing
- Physically or mentally incapable or incompetent

### **Patient accrual**

A medical oncologist will propose the study during a regular hospital visit to each eligible patient. The patient is asked for permission to talk with the investigator directly after their regular appointment. The investigator will then inform him/her about the goal and reason for the study and answer questions. If a patient approves, permission will be asked to use clinical data and collect fecal samples. A patient information letter will be provided. Finally, he or she is asked to sign an informed consent. The patient will have minimal 3 days to decide to participate. The informed consent will be collected (by phone) before or at the start of the first treatment day. The subjects can leave the study at any time for any reason if they wish to do so without any consequences.

### **Study procedure**

The patients follow the regular treatment program initiated by an oncologist of the MUMC+ or Zuyderland Medical Centre. Since treatment schedules of capecitabine (with or without bevacizumab) and TAS-102 slightly differ, both study procedures will be described in the following section.

#### Patients that receive capecitabine (with or without bevacizumab)

Before the start of each treatment cycle, the patient visits the hospital for a regular check up and blood sample collection. On days 1–14, capecitabine therapy 1250 mg/m^2^ is orally administered twice daily, every 3 weeks. Each dose capecitabine should be ingested with water 30 min after a light meal. The total daily dose amount is 2500 mg/m^2^. Dose reductions are allowed following regular institutional practice. The treating medical oncologist is allowed to give additional treatment of the VEGF-inhibitor bevacizumab 7.5 mg/kg, which will be administered intravenously at the day care clinic on day 1 of every cycle. During the second week of the third treatment cycle, a computed tomography (CT)—or magnetic resonance imaging (MRI)—scan will be performed for response monitoring.

In addition to regular treatment, the patient is asked to collect a fecal sample and complete a questionnaire before (T1), during (T2), and after (T3) three treatment cycles with systemic treatment. The patient can collect the fecal samples in the hospital or up to 2 days before hospital visit at home. Sample collection can be performed easily and hygienically with the collection device provided in less than 5 min. If collected at home, the sample should be stored in the freezer (−20 °C) and needs to be transported to the hospital with a cool transport container (Sarstedt) that will be distributed to all patients.

**Fig. 1:**
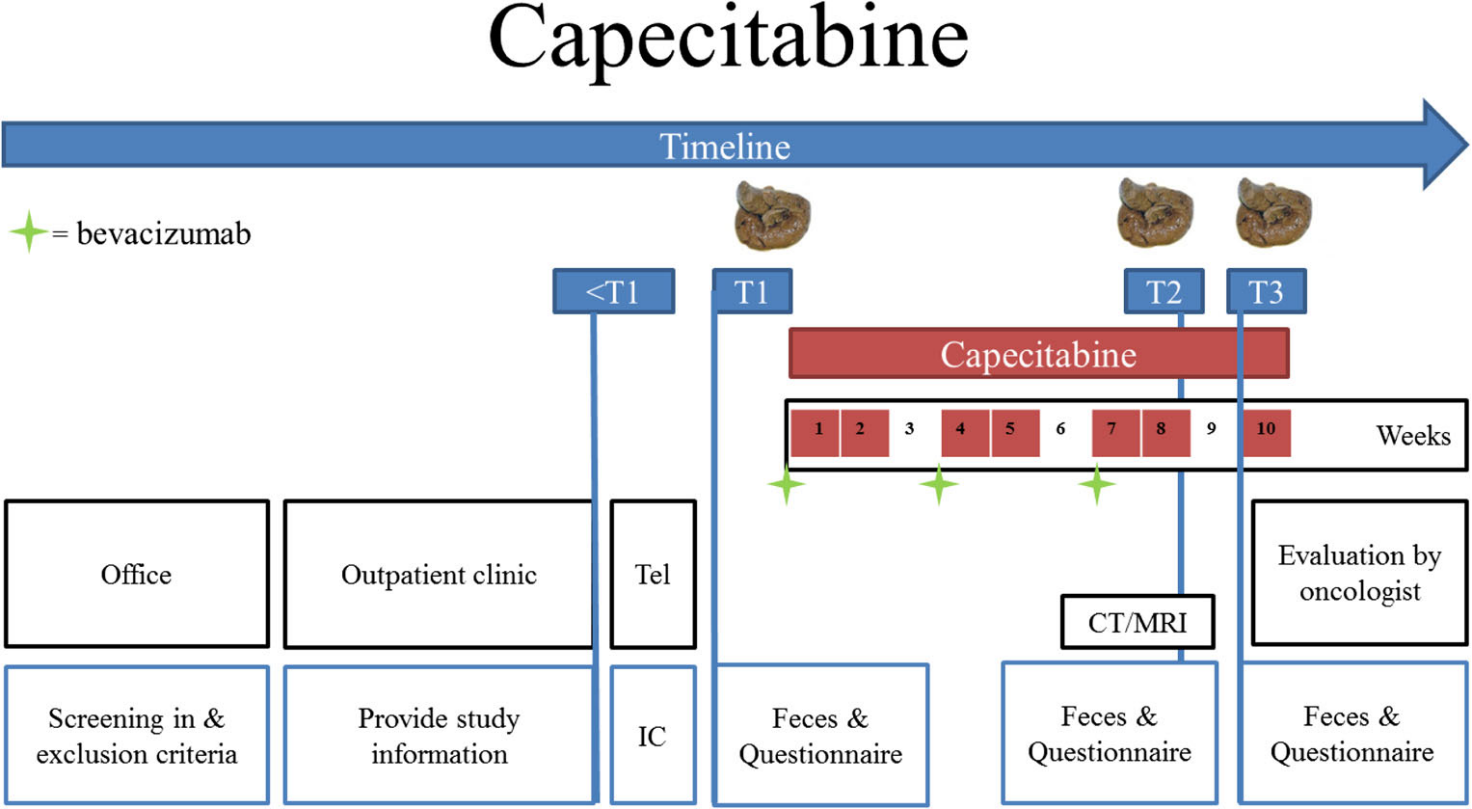
Schematic overview of the study procedure for patients treated with capecitabine (with or without bevacizumab)

During all sample collection moments, patients visit the hospital as outlined in Table 1. The second week of the third treatment cycle with capecitabine (with or without bevacizumab) is chosen to collect the fecal sample and questionnaire during treatment (T2), since patients receive one of the last doses of capecitabine during that treatment cycle. It is expected that during these days, the highest capecitabine concentration and potential chemotoxic effects will be reached. No additional hospital visit is needed for the collection of the fecal sample of T2, since patients visit the hospital for response evaluation with a CT or MRI scan during that week. Figure 1 provides a schematic overview of the study procedure.

**Table 1:** Regular and study-related data collected during different time points for patients treated with capecitabine (with or without bevacizumab)

Table 1	Capecitabine		Cycle 1			Cycle 2			Cycle 3			Cycle 4
		Days										
		<1	1	2–14	15–21	22	23–35	36–42	43	44–56	57–63	64
Regular treatment	Hospital visit	X	X			X			X	X		X
	Bevacizumab		X			X			X			X
	Capecitabine		X	X		X	X		X	X		X
	A + PA	X				X			X			X
	CT/MRI	X								X		
	Laboratory	X				X			X			X
Study-related	Measure point	T1								T2		T3
	Questionnaire	X								X		X
	Fecal sample	X								X		X

*A* anamnesis, *PA* physical examination, *CT* computed tomography, *MRI* magnetic resonance imaging, *X* Proc﻿edure ﻿perfor﻿med﻿, *T1* timepoint 1, *T2* timepoint 2, *T3* timepoint 3, *Cycle* chemotherapy cycle

#### Patients that receive TAS-102

Before the start of each treatment cycle, the patient visits the hospital for a regular check up and blood sample collection. TAS-102 is orally administered (35 mg/m^2^/dose) twice daily on days 1–5 and days 8–12, every 4 weeks [18]. During the third treatment cycle, the patient visits the hospital to analyze response by a CT or MRI scan.

In the same ways as for patients that receive capecitabine, the patients will be asked to collect a fecal sample and complete a questionnaire before (T1), during (T2), and after (T3) three treatment cycles with systemic treatment. Sample collection should be performed as described in the capecitabine group.

**Fig. 2:**
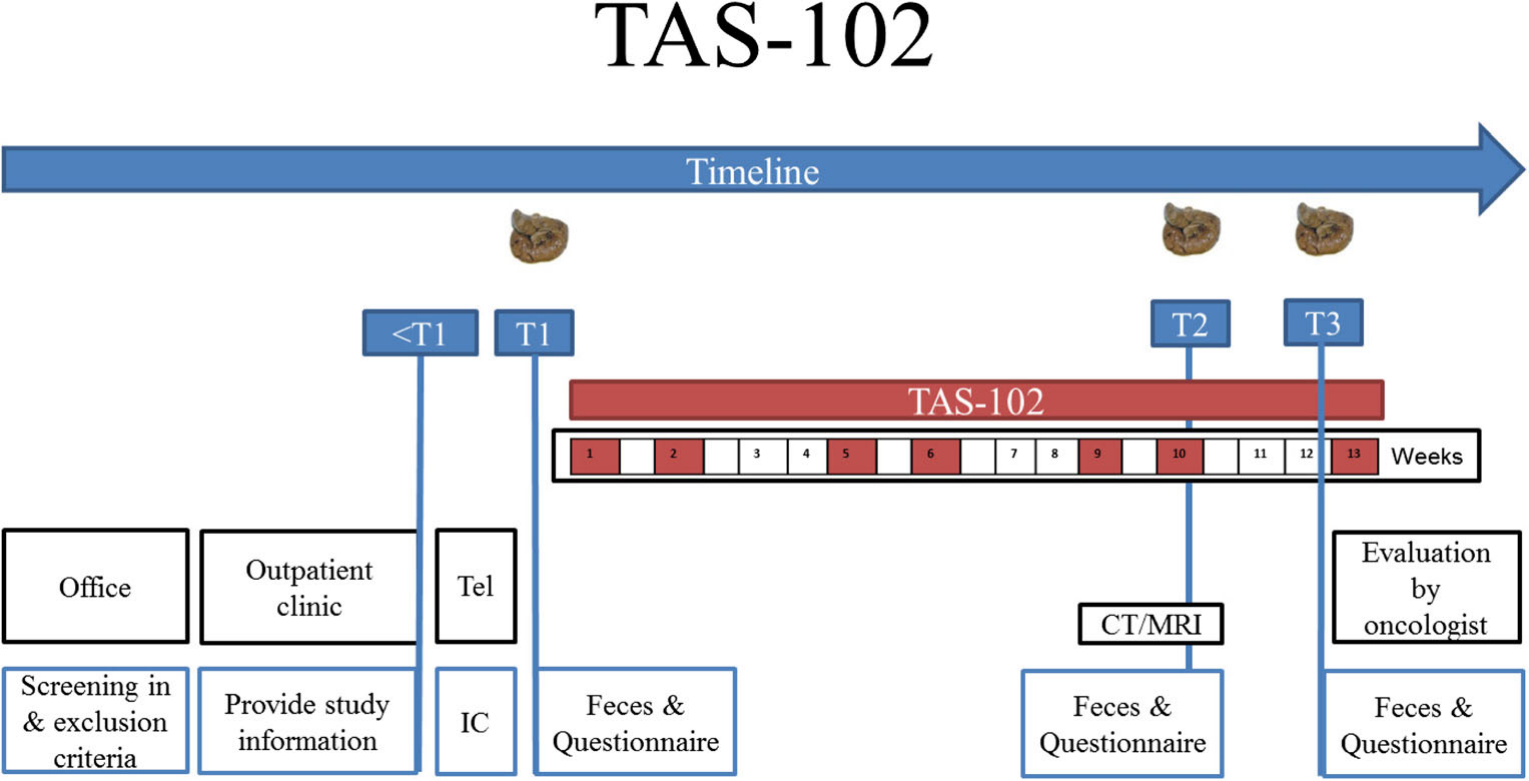
Schematic overview of the study procedure for patients treated with TAS-102

During sample collection of T1 and T3, the patients visit the hospital as outlined in Table 2. The second week of the third treatment cycles with TAS-102 is chosen to collect fecal sample and questionnaire during treatment (T2), since patients receive one of the last doses of TAS-102 during the third treatment cycle. It is expected that during this days the highest TAS-102 concentration and potential chemotoxic effects will be reached. No additional hospital visit is needed since fecal sample of T2 can be collected at home and stored in the freezer for 1 week. The patients can bring both the fecal sample and questionnaire to the hospital during their next hospital visit, which will be approximately 1 week later for response evaluation with a CT or MRI scan. Figure 2 provides a schematic overview of the study procedure.

**Table 2:** Regular and study-related data collected during different time points for patients treated with TAS-102

Table 2	TAS-102		Cycle 1					Cycle 2					Cycle 3					Cycle 4
		Days																
		<1	1–5	6–7	8–12	13–14	15–28	29–33	34–35	36–40	41–42	43–56	57–61	62–63	64–68	69–70	71–84	85
Regular treatment	Hospital visit	X	X					X					X				X	X
	TAS-102		X		X			X		X			X		X			X
	A + PA	X						X					X					X
	CT/MRI	X															X	
	Laboratory	X						X					X					X
Study-related	Measure point	T1													T2			T3
	Questionnaire	X													X			X
	Fecal sample	X													X			X

*A* anamnesis, *PA* physical examination, *CT* computed tomography, *MRI* magnetic resonance imaging, ﻿*X* Proc﻿edure ﻿perfor﻿med﻿, *T1* timepoint 1, *T2* timepoint 2, *T3* timepoint 3, *Cycle* chemotherapy cycle

In general, there will be no risk for the patient, since no intervention or treatment will be initiated. The patients will follow regular treatment program initiated by their oncologist. There will be almost no burden for participating patients in this study. The fecal samples and questionnaires could be collected at home or during hospital visits. Fecal sample collection is very easy with the provided collection device and can be performed hygienically in less than 5 min.

### Data collection and sample handling

After the patient has given informed consent, fecal samples, questionnaires, and data from the patients’ medical record, including chemotherapy dose reduction, will be collected. All data, except fecal samples, will be collected in the case report form (CRF). Personal data will be handled with strict care securing the patients’ privacy. Results will only be used for research purposes, not for clinical purposes. All patients’ CRFs and their fecal samples receive a study code. All coded data will be entered into a secured database and statistically analyzed. The key linking study code to patient data (hospital ID and name) will only be accessible to the investigator. Conform current guidelines, all CRFs, fecal samples, and questionnaires will be stored for a maximum of 15 years for any future studies in line with the current study.

Patient inclusion number:

- ID_MB_CRC_001 to ID_MB_CRC_066

Coding of fecal sample collection collected on T1, T2, and T3:

- T1_FS_MB_CRC_001 to T1_FS_MB_CRC_066
- T2_FS_MB_CRC_001 to T2_FS_MB_CRC_066
- T3_FS_MB_CRC_001 to T3_FS_MB_CRC_066

Once fecal samples arrive in the hospital, samples will be coded and stored at −20 °C before being transported in bulk badges to the Biobank of Maastricht for long time storage at −80 °C. The intestinal microbiota composition and abundance will be analyzed with 16S ribosomal RNA (rRNA) next-generation sequencing. Subsequent quantitative polymerase chain reaction (qPCR) will be conducted to convert relative abundance to absolute abundance.

The response to systemic treatment will be measured with CT or MRI: Tumor response is classified by the Response Evaluation Criteria in Solid Tumors (RECIST) version 1.1 published in January 2009. The following categories are used [19]:

- CR (complete response) = disappearance of all target lesions
- PR (partial response) = 30% decrease in the sum of the longest diameter of target lesions
- PD (progressive disease) = 20% increase in the sum of the longest diameter of target lesions
- SD (stable disease) = small changes that do not meet above criteria

Chemotoxicity will be scored with the Common Terminology Criteria for Adverse Events (CTCAE) version 4.03 [20]. The following criteria will be scored: nausea, vomiting, oral mucositis, diarrhea, constipation, fever, (febrile) neutropenia, peripheral sensory neuropathy, hand-foot syndrome, fatigue, and alopecia.

Severity will be graded from 1 to 5. A Dutch translated version will be used in our study. These data will be obtained from the questionnaires and medical records.

Patient compliance to oral chemotherapy will be registered with a Morisky Medication Adherence Scale (MMAS-08) [21]. The English version is validated. A Dutch translated version will be used in our study.

### Sample size calculation

Since the relation of the microbiota composition with response prediction to systemic treatment in vivo has not been studied before, a power analysis for sample size calculation is not possible. This explorative study will provide much needed data to explore the differences in microbiota composition between patients that will respond and will not respond to systemic treatment with capecitabine (with or without bevacizumab) or TAS-102. Previous studies on microbiota in other fields (obesity and infections) showed that sample size between 12 and 88 patients is sufficient for this kind of explorative studies [2, 6, 22]. In this study, 60 patients are needed for data analysis. Considering 10% drop out, we need to include 66 patients. In MUMC+, 30 patients will meet our in and exclusion criteria each year. In Zuyderland Medical Centre, this will be 40 patients each year. Considering 50% willing to participate, 66 patients can be included within 2 years.

### Data analysis

Microbial analysis of the fecal samples will be achieved by next-generation sequencing using the MiSeq platform. Metagenomic DNA from fecal samples will be isolated using a combination of repeated bead-beating and column-based purification in accordance with the recommendations of the International Human Microbiome Standards consortium [23]. The V3-V4 hypervariable regions of the 16S rRNA gene will be amplified using bar-coded fusion primers and sequenced using MiSeq 300 PE sequencing (∼25,000 reads/sample). This approach has been proven a powerful tool to provide a complete picture of the diversity and relative abundance of complex microbial communities. Subsequent quantitative polymerase chain reaction (qPCR) will be conducted to convert relative abundance to absolute abundance. Although the current project focuses on the taxonomic microbial profiles, the samples are being properly stored to enable future (functional) metagenomic analyses.

### Statistics

For bioinformatic analysis of MiSeq data, the expandable software package Quantitative Insights Into Microbial Ecology (QIIME) will be used [24]. The QIIME integrates many third party tools that have become standard in the field of microbial community analysis (such as tools for chimera checking, denoising, clustering, aligning, classifying, phylogeny reconstruction, and calculation of diversity measures). After quality filtering and chimera checking, reads are clustered into operational taxonomic units (OTUs) against the Greengenes reference database [25]. For all subsequent analyses, we will normalize the count table of OTUs using variant stabilization by the R package DESeq2 [26] to account for differences in sequencing depth between the samples.

The fecal samples will be analyzed by taxonomic composition and alpha- and beta-diversity indices will be calculated. Gut microbiota analysis will include alpha-diversity analysis of OTU richness and evenness within each sample and beta-diversity analysis between samples. The differential abundance of each OTU between responders and non-responders and patient that experience chemotoxicity or not in relation to microbiota changes will be tested using DESeq2. Results will be reported as log2 fold changes and associated adjusted *p* values.

For visualization and exploration of these complex data sets, cluster analysis and ordination (e.g., principal coordinate analysis) will be used. To identify the main variables that affect the bacterial communities, constrained analysis such as distance-based redundancy analysis (db-RDA) will be applied.

To further test the potential clinical relevance of bacterial communities and clinical factors in CRC, we will carry out a random forest analysis combining the OTU abundances and clinical data. The random forests represent a method to correlate metadata with a set of features (OTUs/clinical factors) and are an effective approach for analyzing and interpreting high-dimensional data. Hereto, we will use the R package randomForestSRC [27] and the Boruta algorithm for feature selection. The bootstrapped feature selection will be repeated 1000 times with differing random seeds.

Final data analysis will compare microbiota composition before, during, and after three treatment cycles with systemic therapy between responders and non-responders measured with CT/MRI and compare microbiota composition between patients that experience chemotoxic effects and those who have limited chemotoxic effects measured with the CTCAE criteria. A prediction model will be developed to predict systemic treatment response and/or chemotoxicity based on the patients’ intestinal microbiota composition. The prediction model will be developed by selecting bacteria and their abundance associated with response and/or chemotoxicity.

## Future

In case of metastatic and/or irresectable colorectal cancer, time and quality of life (Qol) are the most important issues. To limit chemotoxicity and prolong and increase effects of systemic treatment, a great need exists to explore the role of the intestinal microbiota. In vitro studies already suggest that intestinal microbiota plays a putative role in colorectal cancer treatment. By exploring the microbiota composition and changes in relation to response and chemotoxicity, the favorable microbiota composition can be detected. In the future, low-burden fecal microbiota transplantation might result in more time and improved Qol for cancer patients.

## Trial status

The national Dutch Research Ethics Committee of Maastricht University Medical Centre+ approved the study protocol in December 2016. Patient inclusion started in March 2017. The total duration of the project will be approximately 2 years.
